# Shall We Amend Our Therapeutics and Our Prognosis?

**Published:** 1904-10

**Authors:** A. M. Linn

**Affiliations:** Des Moines, Iowa


### SHALL WE AMEND OUR THERAPEUTICS AND OUR PROGNOSIS?

BY A. M. LINN, M.D., DES MOINES, IOWA.

Not infrequently medical men are confronted with cures of apparently incurable cases. The best efforts of our therapeutics are often discounted. It is only the novice who fails to recognize the power of Nature to effect remarkable cures. In well selected cases he is frequently disappointed in not obtaining the desired results.

This is not said in disparagement of the splendid cures obtained from proper medication, but rather to bring to view failures in cases where better results ought to be obtained. This is particularly true in the treatment of nervous diseases. An unfavorable prognosis is made in such diseases as transverse myelitis and anterior poleo-myelitis, and our patients are told little can be done. The specialist in this field holds out to them a faint hope that Nature will in her own good time afford some relief. Our therapeutics correspond in such cases with our prognosis. The physician recognizes a serious pathological lesion in such disease and accords no power to Nature to cure such affections.

It is known that in nerve resections for the cure of severe neuralgias the divided ends will hunt industriously for each other, in order to reestablish their functions. Unpromising pathological conditions affecting nerve structures, however, are rarely amended, with or without therapeutic measures. Galvanism, farradism and all forms of electricity have been invoked. Often they have seemed to prove of great value, and again their promised usefulness has ended in failure. The progress made in medical treatment of nerve affections has scarcely kept pace with that in other more promising fields. The hyperplasia produced by the inflammation of nerve structures is apparently beyond the reach of remedial treatment. The adventitious tissue resulting from a systematic lesion of the cord is beyond the reach of ordinary therapeutic means. The loss of function which it occasions is rarely restored in the presence of this confirmed organic change. We complacently fold our hands and proclaim to our patients that the affection is beyond our reach. Palliative treatment may be used to allay irritation; various expedients are useful to alleviate pain; but we are practically hopeless of being able to render any permanent benefit by treatment.

Recently an interesting case was noted in medical prints in which a young lady of Des Moines afflicted with transverse myelitis of several years' standing was relieved. Interest was attracted to this case largely because the subject had been under the most skillful treatment without relief. It is an old saw that "it takes more than one swallow to make a summer." This interesting case represented, however, some unusual features. Experts had agreed without dissenting opinion in a diagnosis of transverse myelitis. The history and symptoms indicated clearly this condition. Treatment at the hands of the most skilled therapeutists had availed nothing. All kinds of manual manipulation had proven no more successful. Something more than a year ago she was placed in the Methodist hospital, and M. A. Berry, a skilled masseur, essayed to cure this apparently incurable case. In response to his persistent efforts new life was awakened in the useless limbs and the insensate nerves resumed, to all appearances, their normal function. In this instance was accomplished what medical experts had proclaimed as impossible—a conclusion which their failure to relieve had only tended to confirm.

Another, a case of anterior poleo-myelitis, after months of care in the hands of a noted specialist in an eastern metropolis, yielded readily to the persistent efforts of this talented masseur.

The traditions of medicine are somewhat averse to the ready recognition of unorthodox means of cure. "The laying on of hands" was recognized in Biblical times, but in our day is sadly out of use. Cures of systematic lesions of the cord by any method of treatment should arouse the attention of therapeutists. Whatever means offer good results in the treatment of serious nerve lesions merit serious consideration. It is our province as physicians to offer to the afflicted every available means of cure. The interesting fact to medical men in this instance is that the cures were effected. Another fact is that by our ordinary method of treatment no such results are usually obtained.

It becomes, then, an important question whether it is not incumbent upon us to amend our therapeutics. Would it not be well to investigate carefully and ascertain whether the stimulation of the circulation and added physical vigor, obtainable by manipulation, can accomplish results like these with any degree of uniformity? To what extent may such methods aid our usual therapeutic means in the relief of serious affections like these?

The writer is somewhat leaning toward the conviction that the medical profession is gradually becoming convinced of the value of manual therapeutics. The near future may note the more frequent employment of the masseur; and indeed, it may not be long until the masseur, as the nurse, will prove an invaluable aid to the physician.

Facts are stubborn things. Facts like the cures mentioned are extremely interesting. Those seriously afflicted with nervous affections, finding no relief from our ordinary methods of treatment, gladly avail themselves of any means which promise a cure. No more pathetic appeals come to the physician than are made by such as these.

The purpose of this brief article is not to offer a criticism of our therapeutics. Never before have medical agencies accomplished so much for the alleviation of human suffering. The future is full of promise. Very much, indeed, is now being done. The optimist sees far greater results in the future. But while the present is accomplishing much, the ultimate is not yet attained. There is no method of treatment which is not by right the property of the medical profession. The near future will probably see us making large use of manual therapeutics for the relief of our otherwise incurable cases.—*Iowa Medical Journal.*

